# Relationship between White Matter Hyperintensities and Hematoma Volume in Patients with Intracerebral Hematoma

**DOI:** 10.14336/AD.2018.0108

**Published:** 2018-12-04

**Authors:** Xuemei Chen, Yuexinzi Jin, Jian Chen, Xin Chen, Xiang Cao, Linjie Yu, Yun Xu

**Affiliations:** ^1^Department of Neurology, Nanjing Drum Tower Hospital Clinical College of Nanjing Medical University, Nanjing 210008, China; ^2^Department of Neurology, Affiliated Drum Tower Hospital, and Jiangsu Key Laboratory for Molecular Medicine, Nanjing University Medical School, Nanjing 210008, China.; ^3^Jiangsu Province Stroke Center for Diagnosis and Therapy, Nanjing 210008, China; ^4^Nanjing Neuropsychiatry Clinic Medical Center, Nanjing 210008, China

**Keywords:** intracerebral hemorrhage, white matter hyperintensities, hematoma, leukoaraiosis, quantitative analysis

## Abstract

The relationship of white-matter hyperintensity (WMH) to intracerebral hemorrhage (ICH) remains unclear. In this retrospective study, we investigated whether the severity and progression of WMH could be related to the hematoma volume and absorption in ICH. 2338 WMH patients with ICH aged≥40 years receiving brain computed tomography (CT) imaging within 12 hours of ICH symptom onset were screened, and 227 patients were included in the final study. The severity and progression of WMH were assessed using the software programs MRICRON and ITK-SNAP on brain magnetic resonance imaging (MRI) and the hematoma volumes and absorption with ITK-SNAP software on CT. We assessed the association of WMH severity with ICH volume in 227 patients at baseline. Totally 183 of 227 patients underwent repeated CT within 14 days of ICH onset. The relationship of WMH severity to ICH absorption was analyzed in 183 patients. Additionally, among all 227 patients, 37 subjected to another MRI before ICH onset were divided into two groups according to WMH progression: non-progression and progression groups. The link between WMH progression and hematoma volume was examined. The ICH volume was significantly larger in patients with the highest WMH scores than in those with the lowest WMH scores. Larger WMH volume was independently associated with larger ICH volume (odds ratio 1.00; 95% CI, 1.00 to 1.00; P = 0.049). There was a trend towards WMH progression being related to ICH volume (P =0.049). Contrastingly, the WMH volume was not linked with hematoma absorption (P = 0.79). In conclusion, we found that greater severity and progression of WMH were associated with larger ICH volume. Our findings suggest that WMH might provide important prognostic information about patients with ICH and may have implications for treatment stratification.

Intracerebral hemorrhage (ICH) is usually caused by the rupture of small penetrating arteries secondary to hypertensive changes or other vascular abnormalities [1-2]. ICH accounts for approximately 10-20% of all stroke cases and is associated with high morbidity and mortality [3-4]. Common factors influencing ICH include hypertension, cigarette smoking, excessive alcohol consumption, decreased low-density lipoprotein (LDL) cholesterol levels, decreased triglyceride (TG) levels and usage of certain drugs [5]. The spontaneous intracerebral hemorrhage results from small vessel disease (SVD) [6]. There are some MRI markers for SVD, like white matter hyperintensity (WMH), lacunar infarctions (LIs), cerebral microbleeds (CMBs) and perivascular spaces (PVS) [7]. WMH, or leukoaraiosis, represents areas of ischemic white matter damage attributed to degenerative changes to small vessels, including intimal hyperplasia, atherosclerosis, lipohyalinosis and amyloidosis [8]. Therefore, WMH might re?ect the vulnerability of individual brains to pathologic insults [9]. Most researches focus on WMH is associated with ischemic stroke and less on ICH. Our previous research shown that WMH progression within the first two years was an independent predictor of LIs development. One previous publication also indicated greater WMH burden was associated with small vessel stroke compared with other ischemic stroke subtypes [10]. However, there are some disputed results on the relationship between WMH and ICH. It was reported that the presence of WMH is an independent risk factor for warfarin-related ICH and ICH after thrombolytic treatment for acute ischemic stroke [11-13]. Severe WMH are associated with larger ICH volumes and hematoma growth [14]. Increased WMH is an independent predictor of worse functional outcomes in patients after spontaneous ICH [15]. But Dr. Sykora showed that WMH were not associated with initial hematoma volume, hematoma growth, or intraventricular extension. MH were associated with poor outcome independently [16]. Recently, Dr. Boulouis also indicated that the volume of WMH was not related to either hematoma volume or expansion in ICH [6]. Here, we test our hypothesis that the severity and progression of WMH are related to hematoma volume and absorption in retrospective study.

**Figure 1.**
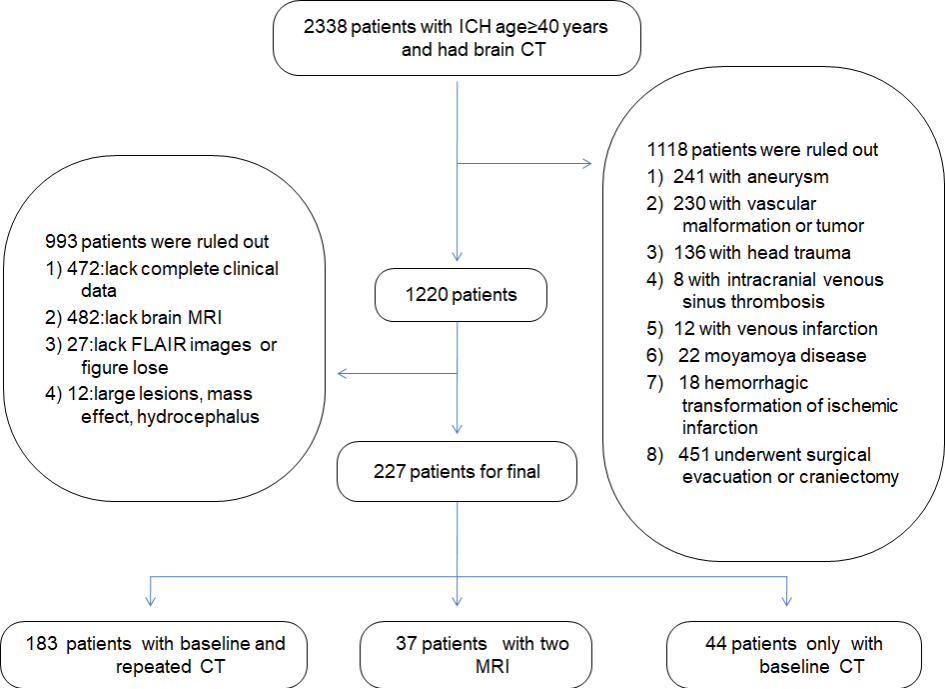
Diagrammatic sketch of the screening process.

## MATERIALS AND METHODS

### Study population

A total of 2338 patients with ICH were studied retrospectively from June 1, 2012, to June 1, 2017, using data from the Drum Tower Hospital imaging center of Nanjing University Medical School. Patients ≥40 years of age were recruited if they had undergone brain computed tomography (CT) within 12 hours of onset of ICH symptoms and received fluid attenuated inversion recovery (FLAIR) and magnetic resonance imaging (MRI) after ICH, and if their baseline clinical and demographic information, including demographic characteristics, medical history, physical examination results and laboratory examination findings, were available. The exclusion criteria included underlying aneurysm, vascular malformation or tumor, head trauma, venous infarction, moyamoya disease, hemorrhagic transformation of ischemic infarction, and previous surgical evacuation or craniectomy. Consequently, 2111 patients were ruled out: complete clinical data were lacking for 472 patients, brain MRI data were not available for 482 patients, FLAIR imaging data were unavailable for 27 patients,241 patients had underlying aneurysm, 230 had vascular malformation or tumor, 136 had head trauma, 8 had intracranial venous sinus thrombosis, 12 had venous infarction, 22 had moyamoya disease, 18 had hemorrhagic transformation of ischemic infarction, and 451 had undergone surgical evacuation or craniectomy, 12 patients were excluded for other reasons. Ultimately, 227 patients, including 44 patients with only baseline CT, 183 patients with baseline and repeated CT and 37 patients with two MRIs were analyzed in this study (Fig. 1). To determine the relation between ICH volume and WMH progression, we selected a total of 37 patients who had undergone another MRI before ICH (the interval between the two examinations was more than one month). The Ethics Committee of Drum Tower Hospital affiliated to Nanjing University Medical School approved this study. The procedures were conducted according to institutional guidelines.

**Figure 2.**
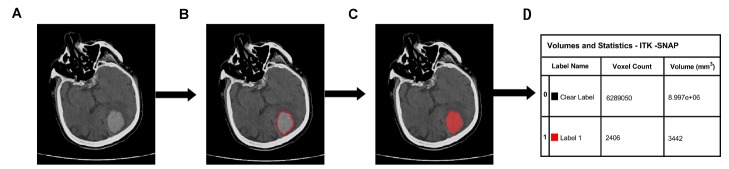
Quantitative steps of ICH volume. (A) Original CT image. (B) The high-density area. (C) A sketch of the high-density area. D) Calculation of ICH volume.

### Risk factors

To assess relevant risk factors at baseline, we examined patient demographic characteristics (age, sex and medical history), history of hypertension, history of diabetes mellitus, presence of coronary heart disease, history of dyslipidemia, and past or present cigarette or alcohol use. Patients underwent a physical examination, systolic and diastolic blood pressure was measured, and laboratory examinations including tests for glucose (random blood glucose RBG, fasting blood glucose FBG, postprandial blood glucose PBG and glycosylated hemoglobin (HbAIc), homocysteine (HCY), total cholesterol (TC), high-density lipoprotein cholesterol (HDL), TG, LDL, apolipoprotein A-I (apoA I), urea nitrogen (BUN), creatinine (Cr), uric acid (UA) and coagulation function were performed.

### Radiologic data

#### CT acquisition and analysis

The hematoma volumes of the 227 patients were measured with ITK-SNAP software (University of Pennsylvania, Philadelphia, USA; www.itksnap.org) at baseline. The baseline scan was limited to 12 hours because most of the hematoma expansion occurred within the following ICH onset after first 24 hours [16]. To determine hematoma absorption, we identified 183 patients who underwent a baseline CT scan within 12 hours and a repeated CT scan within 14 days after ICH onset. We remeasured the repeated ICH volume in 183 patients using ITK-SNAP software. The time window of the repeated CT scan was limited to 14 days after ICH onset because most of the hematoma absorption occurred within 14 days (Fig. 2).

#### MRI acquisition and analysis

MRI scanning was performed on a 1.5-T or 3.0-T scanner. The scanning protocol included a whole-brain T1 magnetization-prepared rapid gradient echo sequence, a transversal T2-weighted turbo spin echo sequence, and FLAIR pulse sequences. The timing of the MRI scan was 7.5±1.5 days after ICH onset.

**Table 1:** Baseline characteristics classified according to trichotomized Fazekas Score.

	Fazekas Score			*P* Value
	1	2	3	

	0-2 (n=79)	3-4 (n=127)	5-6 (n=21)	
Age, y, mean±SD	63.32±13.33	64.73±12.29*	72.10±10.66#	0.02
Men, n (%)	62	92	18	0.52
Past medical history, n (%)				
Hypertension	52	94	16	0.24
Coronary artery disease	10	22	7	0.09
Diabetes mellitus	14	29	4	0.56
Hyperlipidemia	1	6	0	0.19
Smoking, n (%)	22	20	3	0.17
Alcohol, n (%)	16	12	2	0.13
Medications, n (%)				
Use of antiplatelet agents	3	19	1	0.11
Use of anticoagulation	0	4	2	0.25
Use of statin	1	7	0	0.10
NIHSS, mean±SD	4.84±5.50	4.43±4.27*	8.67±7.02#	<0.01
Clinical variables, mean±SD				
SBP, mm Hg	149.76±25.09	152.65±21.15	151.76±16.58	0.66
DBP, mm Hg	87.32±13.59	87.94±16.34	82.90±12.38	0.38
FBG, mM	6.27±2.54	5.83±1.84	7.03±3.43	0.06
RBG, mM	7.18±2.55	7.10±2.59	6.66±1.63	0.72
PBG, mM	8.84±3.30	8.09±2.97	8.43±2.46	0.28
HbAIc, %	5.99±1.12	5.95±1.03	5.79±0.67	0.77
HCY, μM	14.00±4.17	17.35±9.83	14.41±6.10	0.09
TC, mM	4.38±1.19	4.28±0.97	3.69±0.88#	0.03
TG, mM	1.57±1.05	1.36±0.82	1.74±1.34	0.12
LDL, mM	2.39±0.91	2.26±0.69	1.90±0.69#	0.04
HDL, mM	1.01±0.32	1.07±0.39	0.99±0.45	0.46
ApoA I, g/L	1.02±0.25	1.12±0.52	1.01±0.31	0.25
BUN, mM	6.14±4.94	6.55±6.78	6.38±4.14	0.89
Cr, μM	66.8±23.53	80.8±76.98	65.06±17.35	0.20
UA, μM	297.56±129.91	281.57±120.35	255.81±92.68	0.39
Partial thromboplastin time, sec	32.12±32.05	28.82±5.42	27.15±7.79	0.42
International Normalized Ratio	1.26±1.59	1.27±2.24	1.09±0.14	0.91
Fibrinogen, g/L	3.7±2.36	3.3±1.18	4.15±3.72	0.16
D-dimer, mg/L	2.07±3.48	1.27±1.33	1.54±2.55	0.11
Radiologic data				
ICH volume, mL, mean±SD	13.01±13.34	12.23±13.16	21.16±31.21#	<0.01
ICH position, n (%)				0.41
Lobar ICH	25	46	11	
Basal ganglia region ICH	44	67	8	
Brain stem ICH	1	1	0	
Intraventricular ICH	2	0	1	
Multiple ICH	4	6	1	
Cerebellar ICH	4	7	0	

# 1 VS 3 Significant difference (P<0.05),

* 2 VS 3 Significant difference (P<0.05)

#### Radiological diagnosis of WMH

WMHs are hyperintense on T2-weighted or FLAIR sequences but can appear as isointense or hypointense (although less hypointense than CSF) on T1-weighted sequences, depending on the sequence parameters and the severity of the pathological changes [17]. White matter lesions, characterized by bilateral and mostly symmetrical hyperintensities on T2-weighted MRI, are common in older individuals.

### Fazekas scale score

The degree of WMH severity was rated visually on axial FLAIR images using the modified Fazekas scale [18-19], which is the most widely used and validated system for describing WMH severity. This scale divides WMHs into periventricular and deep sites, and periventricular WMHs were graded according to the following patterns: 0 = absent; 1 = caps or pencil-thin lining; 2 = smooth halo; and 3 = irregular periventricular WMH extending into a deep WMH. Deep WMHs were graded according to the following patterns: 0 = absent; 1 = punctate foci; 2 = some confluences of foci; and 3 = large fused areas. The total score (0 to 6) was calculated by adding the scores for periventricular and deep WMHs. Because some hemorrhages obscured the underlying brain parenchyma, we analyzed WMH in the hemisphere without ICH.

**Table 2:** Univariate Risk Factors for Hemorrhage Volume.

	Hemorrhage Volume		*P* Value
	0-50% (n=114)	51-100% (n=113)	
Age, y, mean±SD	64.97±12.73	64.87±12.73	0.95
Men, n (%)	70	64	0.47
Past medical history, n (%)			
Hypertension	82	80	0.85
Coronary artery disease	12	19	0.17
Diabetes mellitus	23	20	0.63
Hyperlipidemia	3	3	0.99
Smoking, n (%)	21	17	0.50
Alcohol, n (%)	14	10	0.40
Medications, n (%)			
Use of antiplatelet agents	11	8	0.49
Use of anticoagulation	1	3	0.31
Use of statin	4	3	0.51
NIHSS, mean±SD	3.92±4.00	6.01±5.91	<0.01*
Clinical variables, mean±SD			
SBP, mm Hg	151.47±24.65	151.65±19.53	0.95
DBP, mm Hg	86.68±15.88	87.87±14.62	0.56
FBG, mM	5.91±2.15	6.27±2.44	0.24
RBG, mM	7.30±2.72	6.86±2.23	0.22
PBG, mM	8.54±3.06	8.23±3.06	0.49
HbAIc, %	6.03±1.12	5.87±0.93	0.32
HCY, μM	15.81±9.38	15.93±6.41	0.93
TC, mM	4.21±1.07	4.29±1.04	0.56
TG, mM	1.47±0.91	1.46±1.02	0.87
LDL, mM	2.25±0.751	2.28±0.81	0.83
HDL, mM	1.06±0.31	1.02±0.42	0.39
ApoA I, g/L	1.12±0.52	1.02±0.31	0.08
BUN, mM	6.03±6.13	6.75±5.79	0.37
Cr, μM	72.04±33.02	76.92±78.09	0.54
UA, μM	285.7±108.19	283.72±148.16	0.91
Partial thromboplastin time, sec	28.44±7.04	31.22±26.89	0.30
International Normalized Ratio	1.40±2.69	1.09±0.34	0.23
Fibrinogen, g/l	3.56±2.56	3.49±1.20	0.79
D-dimer, mg/l	1.06±1.19	2.12±3.19	<0.01*
Radiologic data			
WMH volume, mm^3^, mean±SD	13345.29±10768.43	16510.27±9888.57	0.02*
ICH position, n (%)			0.01*
Lobar ICH	29	53	
Basal ganglia region ICH	71	48	
Brain stem ICH	2	0	
Intraventricular ICH	1	2	
Multiple ICH	5	6	
Cerebellar ICH	6	4	

* Significant difference (P<0.05)

### Quantitative analysis of WMH volume

**Figure 3.**
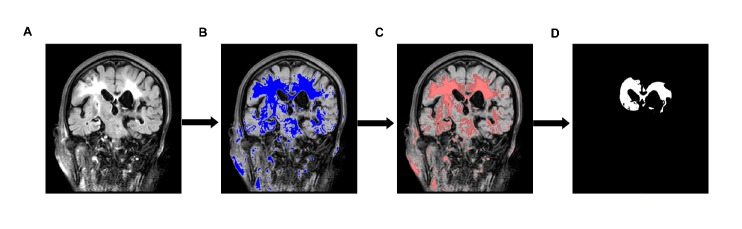
Quantitative steps of WMH volume. (A) Original FLAIR image. (B) The high signal area. C) A sketch of the effective WMH area. (D) Extraction of the effective WMH area.

WMH volumes were quantified using the software programs MRICRON (University of Nottingham School of Psychology, Nottingham, UK; www.mricro.com) and ITK-SNAP (Fig. 3). All scans were checked by visual inspection. First, the MRICRON software was used to extract the effective WMH area, and then, the ITK-SNAP software was used to calculate the WMH volume. Because WMH quantification was affected by hematoma and perihematomal edema, we adopted certain expedients to reduce errors: we excluded cases with very large lesions, mass effects, or hydrocephalus; the time of MRI scan was 7.5±1.5 days after ICH onset to minimize the influence of edema on quantitative WMH measurements.

### Statistical analysis

Mean (standard deviation) or median (interquartile range) values were adopted to determine whether continuous variables had a normal distribution. Frequencies were used to describe categorical variables. Patients were dichotomized according to baseline ICH volume as measured on CT. The proportion of ICH volume absorption was calculated by subtracting the baseline ICH volume from the follow-up ICH volume on CT scans and dividing this value by the baseline ICH volume. The proportion of ICH absorption was dichotomized according to the repeated ICH volume as measured on CT. To determine the relation between ICH and WMH progression, we defined non-progression as a white matter volume on the second measurement less than or equal to that of the first measurement, and we defined progression as a white matter volume on the second measurement greater than that of the first measurement. We used Bonferroni correction to evaluate differences between the two groups. We used Fisher’s exact test to compare dichotomous variables between groups and used the Wilcoxon rank-sum test for comparisons of continuous and ordinal variables. Variables with a P<0.1 based on univariate regression analyses were included in the multivariate logistic regression models. Statistical significance was set at P<0.05.

## RESULTS

### Baseline characteristics classified according to trichotomized Fazekas scale scores

According to the Fazekas scale score, the 227 patients were divided into three groups: Group 1 (score of 0-2), Group 2 (score of 3-4), and Group 3 (score of 5-6). As shown in Table 1, Groups 1 and 2werecompared to Group 3. Patients with higher WMH scores were older and had higher NIHSS scores. When Group 1 was compared to Group 3, patients with higher WMH scores had lower TC levels, lower LDL cholesterol levels, and larger ICH volume (P<0.05). However, when Group 1 was compared to Group 2 or when Group 2 was compared to Group 3, no significant differences were observed.

### Univariate and multivariate logistic regression analyses of risk factors related to ICH volume

As shown in Table 2, the hemorrhage volume was correlated with the NIHSS score, apoA I level, D-dimer level, WMH volume and ICH site (P<0.05). Lobar ICH had a greater ICH volume, but in cases of smaller ICH volume, the most common ICH site was the basal ganglia region. Only WMH volume, ICH position and NIHSS score were independently associated with ICH volume based on the multivariate regression results, as shown in Table 3.

**Table 3:** Multivariate logistic regression analysis of risk factors for ICH volume.

	Odds Ratio	95% CI	*P* Value
NIHSS	1.08	1.01-1.15	0.03*
D-Dimer	1.23	0.99-1.53	0.06
WMH volume	1.00	1.00-1.00	0.049*
ApoA I	0.49	0.20-1.80	0.37
ICH position	1.44	1.09-1.91	0.01*

* Significant difference (P<0.05)

**Table 4:** Analysis of relationship of hematoma volume and WMH progression.

	WMH progress		
	No (n=17)	Yes (n=20)	*P* Value
Age, y, mean±SD	59.24±19.43	57.90±15.60	0.82
Men, n (%)	12	14	0.97
Past medical history, n (%)			
Hypertension	7	12	0.25
Coronary artery disease	0	1	0.26
Diabetes mellitus	3	3	0.83
Hyperlipidemia	1	0	0.21
Smoking, n (%)	1	4	0.19
Alcohol, n (%)	1	5	0.10
Medications, n (%)			
Use of antiplatelet agents	0	1	0.26
Use of anticoagulation	0	0	-
Use of statin	1	0	0.21
Clinical variables, mean±SD			
SBP, mm Hg	136.65±21.92	145.20±19.17	0.21
DBP, mm Hg	77.35±12.61	83.75±10.56	0.10
FBG, mM	5.61±1.75	5.48±1.72	0.82
RBG, mM	7.32±4.11	6.99±2.70	0.80
PBG, mM	8.43±4.09	7.90±2.03	0.67
HbAIc, %	6.07±0.67	6.02±1.29	0.93
TC, mM	4.02±1.29	3.79±0.69	0.57
TG, mM	1.13±0.44	1.37±0.83	0.38
LDL, mM	2.15±0.95	1.98±0.52	0.58
HDL, mM	1.00±0.28	0.93±0.27	0.57
ApoA I, g/L	1.06±0.25	0.98±0.27	0.41
BUN, mM	6.54±3.41	5.56±1.50	0.34
Cr, μM	87.17±31.23	69.29±25.03	0.12
UA, μM	319.00±112.34	297.71±118.94	0.65
Partial thromboplastin time, sec	32.21±5.19	32.73±11.95	0.89
International Normalized Ratio	1.10±0.07	1.14±0.28	0.65
Fibrinogen, g/L	2.85±1.10	3.36±1.11	0.26
D-dimer, mg/L	1.39±1.21	1.20±1.01	0.70
Radiologic data			
ICH volume, ml, mean±SD	7.12±9.19	13.59±10.25	0.049*

* Significant difference (P<0.05)

### Analysis of the relationship between hematoma volume and WMH progression

As shown in Table 4, of all 227 patients, thirty-seven patients who had received two MRIs before ICH onset were analyzed (the interval between the two examinations was more than one month). In this subset of patients, WMH progression was correlated with ICH volume (P<0.05). However, a trend was observed only for the association between WMH progression and hematoma volume because of the low sample size.

### Univariate and multivariate logistic regression analyses of risk factors related to the proportion of hemorrhage absorption

Hemorrhage absorption was correlated with ICH volume, history of hypertension and postprandial blood glucose (PBG). However, no trend of an association between WMH volume and proportion of hematoma absorption was observed (Table 5). After adjusting for other confounding variables that could influence ICH absorption, only high ICH volume was independently associated with a high proportion of ICH absorption based on multivariate regression (Table 6).

## DISCUSSION

The main findings of this study were as follows: 1) there was an independent association between the severity of WMH and the ICH volume in this cohort of patients, according to quantitative WMH measurements and Fazekas scale scores; 2) WMH progression was correlated with increased ICH volume, suggesting that white matter damage might predict ICH volume.

**Table 5:** Univariate analysis of hematoma absorption proportion.

	Hemorrhage Absorption Proportion		*P* Value
	0-50% (n=48)	51-100% (n=63)	
Age, y, mean ±SD	64.35±12.65	65.47±12.42	0.56
Men, n (%)	48	63	0.11
Past medical history, n (%)			
Hypertension	48	91	0.03*
Coronary artery disease	10	12	0.64
Diabetes mellitus	11	24	0.34
Hyperlipidemia	0	3	0.28
Smoking, n (%)	11	21	0.56
Alcohol, n (%)	8	10	0.80
Medications, n (%)			
Use of antiplatelet agents	4	12	0.29
Use of anticoagulation	1	3	0.49
Use of statin	1	4	0.65
NIHSS, mean±SD	5.44±6.00	4.58±4.50	0.28
Clinical variables, mean±SD			
SBP, mm Hg	149.73±26.85	153.32±19.69	0.30
DBP, mm Hg	87.75±15.76	87.88±14.99	0.95
FBG, mM	5.94±2.26	5.88±1.89	0.84
RBG, mM	6.95±2.45	7.31±2.65	0.40
PBG, mM	7.72±2.54	8.79±3.34	0.04*
HbAIc, %	5.87±0.92	6.06±1.15	0.33
HCY, μM	14.74±7.54	17.45±8.94	0.14
TC, mM	4.25±1.06	4.28±0.99	0.82
TG, mM	1.38±1.00	1.39±0.78	0.97
LDL, mM	2.24±0.83	2.31±0.71	0.54
HDL, mM	1.04±0.38	1.07±0.39	0.69
ApoA I, g/L	1.04±0.29	1.11±0.53	0.32
BUN, mM	5.74±2.73	6.40±7.07	0.45
Cr, μM	66.81±27.10	79.78±80.87	0.19
UA, μM	276.62±94.89	272.38±123.87	0.81
Partial thromboplastin time, sec	29.33±5.57	27.99±5.49	0.12
International Normalized Ratio	1.06±0.20	1.29±2.41	0.42
Fibrinogen, g/l	3.49±1.14	3.57±1.87	0.78
D-dimer, mg/l	1.91±2.99	1.41±2.30	0.26
Radiologic data			
WMH volume, mm3, mean±SD	15344.49±10501.54	14939.61.82±9674.11	0.79
Fazakse score, mean±SD	2.06±1.12	2.09±0.97	0.83
ICH volume, mL, mean±SD	16045.12±14055.45	11720.40±12750.35	0.03*
ICH position, n (%)			0.16
Lobar ICH	26	34	
Basal ganglia region ICH	37	65	
Brain stem ICH	1	1	
Intraventricular ICH	--	--	
Multiple ICH	1	7	
Cerebellar ICH	6	3	

* Significant difference (P<0.05)

WMH is a neuroimaging finding of white matter changes and is thought to be a result of ischemic injury and demyelination [20-21]. WMHs are related to ischemic stroke [21-22], ICH, global functional decline [23], and dementia [24]. WMH has been considered to reflect damage to small vessels in periventricular and subcortical areas, and autopsy studies have shown that the pathogenesis of WMH includes myelin pallor, dilatation of perivascular spaces and tissue myelin rarefaction [25-26]. WMH is associated with certain vascular risk factors, such as age, hypertension, diabetes mellitus, dyslipidemia, and smoking [26-28]. As shown in our study, the group with the highest Fazekas scale scores had significantly higher age, NIHSS score, and ICH volume but lower TC and LDL cholesterol levels than the group with the lowest Fazekas scale scores (P<0.05). The more serious the WMH was, the more significant these differences were. The NIHSS is a 15-item neurological function scale used in patients with acute stroke. A higher NIHSS score reflects a worse patient condition. Higher Fazekas scale scores suggest a greater degree of white matter and brain damage, and the Fazekas scale score has a positive correlation with the NIHSS score.

ICH is the second most common subtype of stroke and is a critical disease that usually leads to severe disability or death. Spontaneous ICH has been attributed to the rupture of arteries previously damaged by chronic hypertension. Pathologic changes in ICH include microaneurysms, fibrinoid degeneration and lipohyalinosis. Compared to ischemic stroke, ICH has higher mortality and more severe disability [29]. Therefore, it is important to study the pathophysiology, risk factors, and prognostic factors of ICH. ICH and WMH share several risk factors in common (hypertension, cerebrovascular disease) and may share a common underlying pathological mechanism involving microangiopathy [15]. As a surrogate for cerebral microangiopathy, WMH has been previously associated with increased risk of spontaneous and medication-induced intraparenchymal hemorrhage putatively via changes in vessel wall integrity, disruption of the brain-blood barrier, and loss of tissue density [8, 30, 31]. The extent of this pathology may contribute to more severe hematomas, more frequent hemorrhage growth, and intraventricular extension. In this study, we dichotomized patients by hemorrhage volume, and we found a significant association between the severity of WMH and hemorrhage volume, which is consistent with the results of Lou [14]. Moreover, in the subgroup in which the relationship between WMH progression and ICH volume was analyzed, we found a positive relationship between the progression of white matter changes and larger ICH volumes. These common pathophysiologic features between WMH and ICH and the impact of WMHs on brain density provide mechanistic links accounting for our findings, whereby hypertension or cerebral amyloid angiopathy (CAA)may play a role in the progression of WMH in association with ICH.

NIHSS scores indicated the degree of disease severity and were closely related to the ICH volume. Higher NIHSS scores were associated with larger ICH volumes. As shown in this study. The ICH site was also associated with ICH volume based on multivariate analysis. It is commonly noted that cerebral hemorrhage occurs most frequently in the lobar and basal ganglia regions. In this study, lobar hemorrhage was associated with greater ICH volume. The pathophysiologic basis for our findings and its potential relation to WMH that frequently accompanies CAA require further investigation.

Known poor prognostic factors of ICH include large hematoma volume, hematoma expansion and absorption, intraventricular hemorrhage, infra-tentorial location, older age, and anticoagulation treatment [5]. With regard to acute brain bleeding, a large case-control study showed that extensive white matter lesions were associated with lower Glasgow Coma Scale scores and higher mortality [5]. However, our study did not find a clear relationship between the severity of WMH and ICH absorption, as ICH absorption was related to only the initial ICH volume. Further research is needed to explore the relationship between the burden of WMH and ICH absorption.

**Table 6:** Multivariate Logistic Regression Analysis of Risk Factors for Hematoma Absorption Proportion.

	Odds Ratio	95% CI	*P* Value
Hypertension	0.56	0.24-1.33	0.19
ICH volume	1.00	1.00-1.00	0.02*
PBG	1.12	0.99-1.28	0.08

* Significant difference (P<0.05)

### Limitations

The limitations of our study are as follows. First, this was a single-center, retrospective cohort study design, which should not be extensively relied upon in clinical practice. Future prospective studies should address these limitations. Second, hematoma growth was examined in a small subset of 37 patients. These results, therefore, should be considered preliminary and hypothesis-generating. Further prospective investigations in larger cohorts are needed to confirm the results. Finally, although some measures have been taken to reduce error, the calculated white matter volume was not accurate because the WMH quantification was affected by many factors. Recently, it has been shown that diffusion tensor imaging (DTI) is useful for detecting microstructural abnormalities in white matter. Thus, DTI is expected to supersede conventional methods of WMH measurement in the future [32].

### Conclusions

In conclusion, it was found that the severity and progression of WMH were correlated with ICH volume in patients with ICH. Our findings suggest that WMH may provide important prognostic information about patients with ICH and provide implications for the prevention and treatment of WMH formation or progression. To reduce the occurrence of ICH and improve the functional outcomes of patients after ICH, WMH should be addressed at the early stages of WMH development in clinical practice. Further studies are needed to elucidate the pathophysiologic link between WMH and ICH and to confirm our findings.

## References

[1] Qureshi AI, Mendelow AD, Hanley DF (2009). Intracerebral haemorrhage. Lancet, 373:1632–1644. 19427958 10.1016/S0140-6736(09)60371-8 PMC3138486

[2] Zuo S, Pan P, Li Q, Chen Y, Feng H (2017). White matter injury and recovery after hypertensive intracerebral hemorrhage. Biomed Res Int. 2017:6138424. 28680884 10.1155/2017/6138424 PMC5478825

[3] Feigin VL, Lawes CM, Bennett DA, Barker-Collo SL, Parag V (2009). Worldwide stroke incidence and early case fatality reported in 56 population-based studies: A systematic review. Lancet Neurol, 8:355–369. 19233729 10.1016/S1474-4422(09)70025-0

[4] Sacco S, Marini C, Toni D, Olivieri L, Carolei A (2009). Incidence and 10-year survival of intracerebral hemorrhage in a population-based registry. Stroke, 40:394–399. 19038914 10.1161/STROKEAHA.108.523209

[5] An SJ, Kim TJ, Yoon BW (2017). Epidemiology, risk factors, and clinical features of intracerebral hemorrhage: An update. J Stroke, 19:3–10. 28178408 10.5853/jos.2016.00864 PMC5307940

[6] Boulouis G, van Etten ES, Charidimou A, Auriel E, Morotti A, Pasi M, et al (2016). Association of key magnetic resonance imaging markers of cerebral small vessel disease with hematoma volume and expansion in patients with lobar and deep intracerebral hemorrhage. JAMA Neurol, 73:1440–1447. 27723863 10.1001/jamaneurol.2016.2619 PMC5584595

[7] Wardlaw JM, Smith EE, Biessels GJ, Cordonnier C, Fazekas F, Frayne R, et al (2013). Neuroimaging standards for research into small vessel disease and its contribution to ageing and neurodegeneration. Lancet Neurol, 12:822–838. 23867200 10.1016/S1474-4422(13)70124-8 PMC3714437

[8] Pantoni L, Garcia JH (1997). Pathogenesis of leukoaraiosis: A review. Stroke, 28:652–659. 9056627 10.1161/01.str.28.3.652

[9] Lee SH, Kim BJ, Ryu WS, Kim CK, Kim N, Park BJ (2010). White matter lesions and poor outcome after intracerebral hemorrhage: A nationwide cohort study. Neurology, 74:1502–1510. 20458066 10.1212/WNL.0b013e3181dd425a

[10] Rost NS, Rahman RM, Biffi A, Smith EE, Kanakis A, Fitzpatrick K, et al (2010). White matter hyperintensity volume is increased in small vessel stroke subtypes. Neurology, 75:1670–1677. 21060091 10.1212/WNL.0b013e3181fc279a PMC3033608

[11] Folsom AR, Yatsuya H, Mosley TH, Jr., Psaty BM, Longstreth WT Jr, (2012). Risk of intraparenchymal hemorrhage with magnetic resonance imaging-defined leukoaraiosis and brain infarcts. Ann Neurol, 71:552–559. 22522444 10.1002/ana.22690 PMC3377969

[12] Curtze S, Haapaniemi E, Melkas S, Mustanoja S, Putaala J, Sairanen T, et al (2015). White matter lesions double the risk of post-thrombolytic intracerebral hemorrhage. Stroke, 46:2149–2155. 26111888 10.1161/STROKEAHA.115.009318

[13] Smith EE, Rosand J, Knudsen KA, Hylek EM, Greenberg SM (2002). Leukoaraiosis is associated with warfarin-related hemorrhage following ischemic stroke. Neurology, 59:193–197. 12136056 10.1212/wnl.59.2.193

[14] Lou M, Al-Hazzani A, Goddeau RP, Jr., Novak V, Selim M (2010). Relationship between white-matter hyperintensities and hematoma volume and growth in patients with intracerebral hemorrhage. Stroke, 41:34–40. 19926840 10.1161/STROKEAHA.109.564955 PMC4821198

[15] Caprio FZ, Maas MB, Rosenberg NF, Kosteva AR, Bernstein RA, Alberts MJ, et al (2013). Leukoaraiosis on magnetic resonance imaging correlates with worse outcomes after spontaneous intracerebral hemorrhage. Stroke, 44:642–646. 23391853 10.1161/STROKEAHA.112.676890

[16] Sykora M, Herweh C, Steiner T (2017). The association between leukoaraiosis and poor outcome in intracerebral hemorrhage is not mediated by hematoma growth. J Stroke Cerebrovasc Dis, 26:1328–1333. 28237126 10.1016/j.jstrokecerebrovasdis.2017.02.003

[17] Brott T, Broderick J, Kothari R, Barsan W, Tomsick T, Sauerbeck L, et al (1997). Early hemorrhage growth in patients with intracerebral hemorrhage. Stroke, 28:1–5. 8996478 10.1161/01.str.28.1.1

[18] Fazekas F, Chawluk JB, Alavi A, Hurtig HI, Zimmerman RA (1987). MR signal abnormalities at 1.5 T in alzheimer's dementia and normal aging. AJR Am J Roentgenol, 149:351–356. 3496763 10.2214/ajr.149.2.351

[19] Boutet C, Rouffiange-Leclair L, Schneider F, Camdessanche JP, Antoine JC, Barral FG (2016). Visual assessment of age-related white matter hyperintensities using flair images at 3 T: Inter- and intra-rater agreement. Neurodegener Dis, 16:279–283. 26646220 10.1159/000441420

[20] Bivard A, Cheng X, Lin LT, Levi C, Spratt N, Kleinig T, et al (2016). Global white matter hypoperfusion on ct predicts larger infarcts and hemorrhagic transformation after acute ischemia. CNS Neurosci Ther, 22:238–243. 26775830 10.1111/cns.12491 PMC6492900

[21] Ay H, Arsava EM, Rosand J, Furie KL, Singhal AB, Schaefer PW, et al (2008). Severity of leukoaraiosis and susceptibility to infarct growth in acute stroke. Stroke, 39:1409–1413. 18340093 10.1161/STROKEAHA.107.501932

[22] Arsava EM, Rahman R, Rosand J, Lu J, Smith EE, Rost NS, et al (2009). Severity of leukoaraiosis correlates with clinical outcome after ischemic stroke. Neurology, 72:1403–1410. 19380699 10.1212/WNL.0b013e3181a18823 PMC2677507

[23] Inzitari D, Pracucci G, Poggesi A, Carlucci G, Barkhof F, Chabriat H, et al (2009). Changes in white matter as determinant of global functional decline in older independent outpatients: Three year follow-up of ladis (leukoaraiosis and disability) study cohort. BMJ, 339: b2477. 19581317 10.1136/bmj.b2477 PMC2714680

[24] Boulouis G, Charidimou A, Auriel E, Haley KE, van Etten ES, Fotiadis P, et al (2016). Intracranial atherosclerosis and cerebral small vessel disease in intracerebral hemorrhage patients. J Neurol Sci, 369:324–329. 27653918 10.1016/j.jns.2016.08.049

[25] Debette S, Markus HS (2010). The clinical importance of white matter hyperintensities on brain magnetic resonance imaging: Systematic review and meta-analysis. BMJ, 341:c3666. 20660506 10.1136/bmj.c3666 PMC2910261

[26] Chowdhury MH, Nagai A, Bokura H, Nakamura E, Kobayashi S, Yamaguchi S (2011). Age-related changes in white matter lesions, hippocampal atrophy, and cerebral microbleeds in healthy subjects without major cerebrovascular risk factors. J Stroke Cerebrovasc Dis, 20:302–309. 20634092 10.1016/j.jstrokecerebrovasdis.2009.12.010

[27] Luo Y, Li J, Zhang J, Xu Y (2014). Low HDL cholesterol is correlated to the acute ischemic stroke with diabetes mellitus. Lipids Health Dis, 13:171. 25395241 10.1186/1476-511X-13-171 PMC4240873

[28] Helenius J, Mayasi Y, Henninger N (2017). White matter hyperintensity lesion burden is associated with the infarct volume and 90-day outcome in small subcortical infarcts. Acta Neurol Scand, 135:585–592. 27573379 10.1111/ane.12670 PMC5332524

[29] Keep RF, Hua Y, Xi G (2012). Intracerebral haemorrhage: Mechanisms of injury and therapeutic targets. Lancet Neurol, 11:720–731. 22698888 10.1016/S1474-4422(12)70104-7 PMC3884550

[30] Starr JM, Wardlaw J, Ferguson K, MacLullich A, Deary IJ, Marshall I (2003). Increased blood-brain barrier permeability in type ii diabetes demonstrated by gadolinium magnetic resonance imaging. J Neurol Neurosurg Psychiatry, 74:70–76. 12486269 10.1136/jnnp.74.1.70 PMC1738177

[31] Farnaz Farokhian, Chunlan Yang1, Iman Beheshti, Hiroshi Matsuda, Shuicai Wu (2017). Age-Related Gray and White Matter Changes in Normal Adult Brains. Aging Dis, 8(6): 899–909. 29344423 10.14336/AD.2017.0502 PMC5758357

[32] Tamura Y, Araki A (2015). Diabetes mellitus and white matter hyperintensity. Geriatr Gerontol Int, 15 Suppl 1:34–42. 10.1111/ggi.12666 26671155

